# Lab Launches—New Leaders, New Discoveries

**DOI:** 10.1128/msystems.00609-26

**Published:** 2026-06-09

**Authors:** Ira J. Blader, Ashley Shade

**Affiliations:** 1 Department of Biomedical Sciences and Pathobiology, Virginia Maryland College of Veterinary Medicine, Virginia Tech 229659, Blacksburg, Virginia, USA; 2 Université Lyon 1, CNRS, INRAE, LEM, UMR 5557, UMR 1418 133614 , VIlleurbanne, France

**Keywords:** lab launches, new leaders, new discoveries

## EDITORIAL

In the United States and some other countries, many small businesses mark their beginnings by posting their first earned dollar (or whatever relevant currency) from their very first transaction on a public-facing wall. It is an act of community celebration that allows these new businesses, together with their staff and customers, to highlight their first act of productivity as an independent entity.

**Fig 1:**
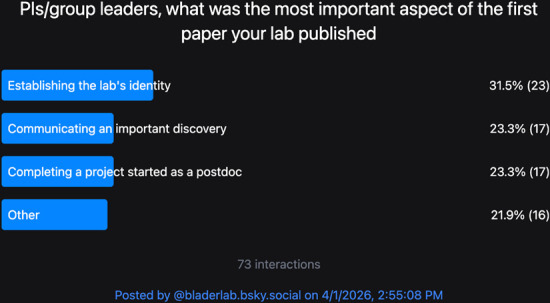
Results of BlueSky poll.

Similarly, in science, peer-reviewed research papers are a statement of productivity and a form of currency. Many labs create traditions to commemorate a paper’s acceptance for publication, with examples including champagne toasts and shared celebratory meals. It is no surprise that many principal investigators (PIs) and team leaders have a special spark in their eyes when discussing that very first primary research article published by their new, independent group. Why is that? We asked that exact question via a BlueSky poll (https://tinyurl.com/sybduf3b), and the responses were, not surprisingly, as diverse as the science itself (Fig. 1). For some, it served as their first new discovery, while for others, it signified their closing of the final chapter as a trainee. For many, the first paper serves as their way to announce that their labs are “open for business” and represents a hard-earned, carefully prepared, and highest-quality research product that defines their new lab’s unique focus and identity.

As editors in chief, we love reading labs’ first papers and are especially honored when new PIs entrust us to peer review and publish them on their behalf. But we realized two things. First, scientists lack that proverbial community-facing wall to display their groups’ first independent research article for the rest of the world to see and celebrate. Second, these papers may be quickly forgotten by the community because research remains forward-looking and has become exceedingly fast-paced. With growing pressure to publish in quantity, sometimes at the expense of quality, we worry that these first research articles, often exemplars of meticulous, rigorous, and innovative work, are getting lost in the fray of high-speed publication pressures.

We believe that honoring critical firsts like this is important and warrants the same care and significance as other research career milestones. This led us to propose a new way that we, as society journals that support our research community, could serve. We want to offer the equivalent of the “wall” that empowers our microbial science community to not only elevate and celebrate these first papers but also sustain them in our collective memory by offering a place where they can be easily found, discussed, and re-discussed in changing contexts as research advances.

Thus, we were compelled to create a new special collection named “Lab Launches—New Leaders, New Discoveries.” Here, new PIs have a celebrated public place to “hang” their first paper for the world to see, not only when they are first published but also when they are returned to and read many times. Thus, our goal is for this collection to serve the microbial science community as a resource to see how our junior colleagues contributed early in their independent careers to solving pressing research questions and set them up to continue to do so. We aspire that this community resource will also support equity in lab debuts by offering all new PIs a shared, open-access forum, regardless of their nationality or geographic location. We invite new PIs and group leaders to submit their first independent papers to *mSphere* and *mSystems* and contribute to this collection; they can do so by indicating that they would like their article included in the Lab Launches collection in the cover letter of their manuscript submission.

New PIs and team leaders are proud of their first research. Here at *mSphere* and *mSystems*, we are, too. Let’s celebrate these new lab milestones together!

## EDITOR'S NOTE

**Ed. Note:** This editorial is being published by *mSphere* and *mSystems*.

